# External Validation and Comparative Performance of the T.O.HO. and S.T.O.N.E. Scoring Systems for Predicting Stone-Free Outcomes Following Flexible Ureteroscopy: Toward Personalized Preoperative Counseling

**DOI:** 10.3390/jpm15100477

**Published:** 2025-10-02

**Authors:** Yuka Sugizaki, Takanobu Utsumi, Rino Ikeda, Naoki Ishitsuka, Takahide Noro, Yuta Suzuki, Shota Iijima, Takatoshi Somoto, Ryo Oka, Takumi Endo, Naoto Kamiya, Hiroyoshi Suzuki

**Affiliations:** Department of Urology, Toho University Sakura Medical Center, Sakura 285-8741, Japan; yuuka.kizuki@med.toho-u.ac.jp (Y.S.); rino.ikeda@med.toho-u.ac.jp (R.I.); naoki.ishitsuka@med.toho-u.ac.jp (N.I.); takahide.noro@med.toho-u.ac.jp (T.N.); yuta.suzuki@med.toho-u.ac.jp (Y.S.); shouta.iijima@med.toho-u.ac.jp (S.I.); takatoshi.soumoto@med.toho-u.ac.jp (T.S.); ryou.oka@med.toho-u.ac.jp (R.O.); takumi.endou@med.toho-u.ac.jp (T.E.); naoto.kamiya@med.toho-u.ac.jp (N.K.); hiroyoshi.suzuki@med.toho-u.ac.jp (H.S.)

**Keywords:** personalized medicine, individualized risk prediction, calibration, decision curve analysis, flexible ureteroscopy, nomogram, stone-free rate

## Abstract

**Background/Objectives**: The attainment of a stone-free (SF) condition is a fundamental indicator of successful outcomes after flexible ureteroscopy (fURS) for urinary stone disease. External confirmations of preoperative scores remain limited. We externally validated the T.O.HO. and S.T.O.N.E. scores in an independent Japanese cohort and examined calibration, decision curve utility, and threshold-guided use to support personalized planning. **Methods**: We retrospectively analyzed 361 consecutive patients treated with fURS from March 2018 to August 2023. Postoperative SF status was defined as the absence of residual calculi greater than 2 mm on non-contrast computed tomography performed within three months of surgery. Independent determinants of SF were identified using multivariable logistic regression, predictive performance was quantified by receiver operating characteristic analyses with DeLong’s test, and model calibration and decision curve analysis were additionally assessed. **Results**: Among the 361 patients, 255 (70.6%) achieved an SF state. A larger stone diameter, the presence of lower-pole calculi, and preoperative pyuria (positive urine WBC) were significant independent predictors of residual fragments. T.O.HO. demonstrated superior discrimination (AUC 0.86) compared with S.T.O.N.E. (AUC 0.77; *p* < 0.01) and surpassed individual predictors. Both scores showed acceptable calibration. Decision curve analysis demonstrated higher net benefit for T.O.HO. across clinically relevant thresholds. We provide clinically useful cut-offs (e.g., T.O.HO. ≤5: high SF probability; 6: trade-off discussion; ≥7: higher residual risk) to align actions with patient priorities. **Conclusions**: Beyond discrimination, a calibrated, threshold-aware use of T.O.HO. enables personalized preoperative counseling and shared decision-making, potentially reducing unnecessary staging and enhancing routine fURS planning.

## 1. Introduction

Urolithiasis is a prevalent urological disorder, with a lifetime incidence estimated at approximately 10–15% worldwide [1,2,3]. Due to its chronic nature and high recurrence rate, it imposes a substantial socioeconomic burden on healthcare systems [4,5]. Flexible ureteroscopy (fURS) is now firmly established as a primary therapeutic option for stones of the upper urinary tract—encompassing both ureteral and renal calculi—owing to its minimally invasive approach, relatively low complication rate, and favorable stone-free (SF) outcomes, particularly in cases where shockwave lithotripsy proves less effective [5,6]. Nevertheless, despite significant technological advances in laser systems, digital ureteroscopes, and stone retrieval devices, the SF rate after fURS remains variable and largely dependent on patient-specific and stone-related characteristics.

Accurately predicting the likelihood of achieving a SF state prior to surgery is of paramount importance for guiding clinical decision-making, aligning patient expectations, and optimizing perioperative planning [7,8]. Several risk stratification instruments, such as nomograms, have thus been developed with the aim of estimating the chance of achieving SF after fURS. Notable examples include the R.I.R.S. score [9], the H.L.P.E.S. score [10], the S.T.O.N.E. score [11], and more recently, the T.O.HO. score [12].

The S.T.O.N.E. and T.O.HO. scores are particularly appealing for their simplicity and ease of use in routine preoperative assessment [11,12]. The S.T.O.N.E. score, introduced by Molina and colleagues in 2014, incorporates five components: stone size, stone location, degree of urinary tract obstruction, number of stones, and computed tomography (CT) Hounsfield unit (HU) evaluation [11]. In contrast, the T.O.HO. score, proposed by Hori and colleagues in 2020, was developed from retrospective data at a single Japanese institution and comprises three objective, readily assessable preoperative parameters: stone length, stone location, and HU evaluation based on CT HU measurements [12]. Although both scoring systems aim to stratify risk using preoperative imaging data, they differ in complexity and constituent variables.

In their original studies, both the S.T.O.N.E. and T.O.HO. scores demonstrated excellent predictive accuracy through internal validation. However, subsequent external validation studies have yielded favorable or unfavorable results [13,14]. Therefore, further validation in independent cohorts with diverse patient demographics, surgical practices, and institutional protocols is critical to assess their generalizability [15]. Comparative evaluation of both scoring systems within the same patient cohort may offer deeper insight into their relative clinical applicability.

Recognizing the persistent need for simple and reproducible risk-assessment tools, we performed an external validation and comparative evaluation of the predictive performance of the S.T.O.N.E. and T.O.HO. scoring systems in a retrospective cohort of patients undergoing fURS for ureteral and/or renal calculi. Beyond average discrimination, personalized stone surgery requires well-calibrated individual probabilities, explicit clinical thresholds that translate predictions into actions, and transparent net benefit at the point of care. Accordingly, we not only externally validated T.O.HO. and S.T.O.N.E., but also assessed calibration and decision curve utility and operationalized threshold-guided use to support shared decision-making in routine fURS planning.

## 2. Materials and Methods

### 2.1. Study Design and Population

This investigation was designed as a retrospective cohort analysis at Toho University Sakura Medical Center, a tertiary referral institution with extensive experience in managing stone disease of the urinary tract. Consecutive patients undergoing fURS for ureteral or renal calculi between March 2018 and August 2023 were included. Inclusion: Adults undergoing fURS as the index procedure with (i) preoperative non-contrast computed tomography (NCCT) and (ii) postoperative NCCT within 3 months. Postoperative NCCT within 3 months was required for inclusion [16,17]. Exclusion: Absence of preoperative NCCT; absence of postoperative NCCT within 3 months; index procedures other than fURS; data-quality deficiencies; bilateral ureteral stones requiring staged or multiple procedures. Same-session bilateral procedures were analyzed at the renal-unit level; patients who underwent retreatment within 3 months were adjudicated as not SF at the index session.

### 2.2. Operative Details: Laser Platforms, Ureteroscopes, and Accessory Devices

All procedures employed holmium: YAG laser platforms, predominantly the Lumenis Pulse 120H high-power system and, in a minority of cases, the VersaPulse Select 30 W (Lumenis Be Ltd., Yokneam, Israel). A 365-µm fiber was used for ureteral stones and a 200-µm fiber for renal stones; when both ureteral and renal calculi were present, the 200-µm fiber was preferred. The primary lithotripsy approach was fragmentation, with adjunctive dusting for small residual fragments. Active fragment retrieval was performed using basket devices (Zero Tip™; Boston Scientific Corporation, Marlborough, MA, USA; Dormia^®^ No-Tip; Coloplast A/S, Humlebæk, Denmark) when clinically indicated.

Initial ureteral access was obtained with a rigid ureteroscope (K27001L; Karl Storz, Tuttlingen, Germany). Lithotripsy was performed with flexible ureteroscopes, including reusable models (Flex-Xc, Flex-X2; Karl Storz SE & Co. KG, Tuttlingen, Germany) and, in selected cases, a single-use disposable scope (WiScope; OTU Medical, San Jose, CA, USA). A ureteral access sheath (Navigator™ HD, 11/13 or 13/15 Fr; or ReTrace™, 10/12 or 12/14 Fr; Boston Scientific Corporation, Marlborough, MA, USA) was used routinely. Manual hand-pump irrigation with room-temperature saline was employed; no pressurized or automated irrigation systems were used.

### 2.3. Data Collection

To delineate factors influencing postoperative SF outcomes, comprehensive clinical and imaging information was retrieved from institutional electronic medical records. As well as relevant comorbid conditions and perioperative parameters, extracted variables encompassed demographic characteristics (sex, age, and body mass index [BMI]), Eastern Cooperative Oncology Group-Performance Status (ECOG-PS), comorbidities (diabetes mellitus, hyperparathyroidism, history of urolithiasis), medication use (e.g., α1-blockers, anticoagulants, corticosteroids), and pre-stenting status. Laboratory and urinary parameters included white blood cell (WBC) count, hemoglobin (Hb), platelet count, C-reactive protein (CRP), hemoglobin A1c (HbA1c), urine pH, urine WBC count, and urine nitrite levels. The evaluation of chronic kidney disease (CKD) was performed using the estimated glomerular filtration rate (eGFR). The eGFR was derived from serum creatinine values according to the revised Japanese equations [18]. For this study, CKD was defined as an eGFR below 60 mL/min/1.73 m^2^, representing each patient’s baseline chronic renal function [18].

Detailed stone characteristics were recorded, including laterality, number, maximum diameter, total stone length of all stones, location (ureteral and renal), and mean NCCT attenuation value of stones. Hydronephrosis grade and solitary kidney status were evaluated on NCCT imaging. For radiologic variables, the average NCCT attenuation of both ureteral and renal calculi was quantified using the hospital’s Picture Archiving and Communication System. The degree of hydronephrosis was graded according to the hydronephrosis classification system: Grade I, dilatation confined to the renal pelvis; Grade II, enlargement of the renal pelvis accompanied by involvement of several calyces; Grade III, marked expansion of the pelvis and all calyces; and Grade IV, pronounced pelvic and calyceal dilatation with associated thinning of the renal cortex [19].

### 2.4. Definition of Stone-Free Status

A patient was considered SF when postoperative NCCT performed within 3 months showed no residual fragments exceeding 2 mm in size [11]. This definition is consistent with previous reports evaluating outcomes after ureteroscopy using NCCT-based assessment [20].

All patients followed a standardized postoperative care pathway, including ureteral stent placement and routine follow-up protocols. They underwent postoperative NCCT between 1 and 3 months after surgery. No alternative imaging modalities were used for outcome adjudication. NCCT scans were independently reviewed by two board-certified urologists blinded to preoperative clinical and operative data; any discrepancies were resolved by consensus.

### 2.5. Scoring Systems

The total T.O.HO. score was calculated as originally outlined by Hori and colleagues [21]. The score components **(**stone length, stone location, and HU of stone**)** were extracted directly from the dataset, and the final score was recorded in the dataset.

The S.T.O.N.E. score was calculated as originally outlined by Molina and colleagues [11]. It consists of five parameters: stone size, stone location, degree of the urinary obstruction, number of stones, and evaluation of HU. Each parameter was assigned a point value, and the total score was derived by summing these components. All variables were extracted from preoperative NCCT images and recorded in the study dataset.

### 2.6. Statistical Analysis

We analyzed complete baseline information from 361 Japanese patients who underwent fURS for upper urinary tract calculi to explore clinical and radiological factors influencing SF outcomes. Initially, univariable testing was conducted to evaluate associations between each candidate parameter and SF status. Continuous data were assessed with either *t*-tests or Mann–Whitney U-tests depending on distribution, whereas categorical variables were examined using χ^2^ tests or Fisher’s exact test when appropriate. Variables demonstrating statistical significance in these univariable assessments were subsequently entered into multivariable logistic regression to determine independent predictors of SF status. A priori, composite scores were excluded from the multivariable model to avoid collinearity with their components; the included covariates were maximum stone size (mm), lower-pole location, urine WBC positivity, a number of stones ≥3, hydronephrosis grade (None–Grade IV), and mean HU.

To evaluate model performance, receiver operating characteristic (ROC) curves were generated for both the S.T.O.N.E. and T.O.HO. scoring systems, and the area under the curve (AUC) was calculated as a measure of discrimination. In addition, the AUC of the T.O.HO. score was compared against those of individual predictors from univariable analyses using DeLong’s test [15]. For each score, we performed logistic recalibration, reporting calibration-in-the-large (intercept) and calibration slope, accompanied by locally estimated scatterplot smoothing (LOESS)-smoothed calibration plots. Decision curve analysis (DCA) quantified net benefit across threshold probabilities of 0.20–0.60. For each score, we selected two clinically meaningful cut-offs: (i) the Youden’s J cut-off (the threshold that maximizes sensitivity + specificity − 1) and (ii) a high-sensitivity cut-off (the smallest threshold with sensitivity ≥ 0.85). Because higher raw scores indicate greater risk of not being SF, patients were classified as SF when the score was ≤the cut-off. For each cut-off we reported sensitivity, specificity, positive predictive value (PPV), and negative predictive value (NPV), calculated from the 2 × 2 confusion matrix and interpreted in the context of the cohort’s SF prevalence.

Statistical significance was defined as a two-sided *p* value < 0.05. All analyses were performed using the JMP Pro 17 Academic Suite (SAS Institute Inc., Cary, NC, USA) (https://www.jmp.com/en/software (accessed on 24 July 2025)) and EZR software (https://www.jichi.ac.jp/saitama-sct/SaitamaHP.files/statmedEN.html (accessed on 24 July 2025), Saitama Medical Center, Jichi Medical University, Saitama, Japan; version 1.68) [22].

### 2.7. Ethical Considerations

The study protocol was approved by the Ethics Committee of Toho University Sakura Medical Center (Approval No. S24084_S23020). Because this investigation was retrospective, the requirement for individual informed consent was waived by the ethics committee. Details of the study protocol were made publicly available on the institution’s website to provide patients with the option to decline participation. All aspects of the research were conducted in accordance with the ethical standards of the Declaration of Helsinki.

## 3. Results

### 3.1. Patient Characteristics

In the final analysis, 361 individuals who underwent fURS were evaluated. The SF rate, determined by the absence of residual fragments larger than 2 mm on NCCT within 3 months after surgery, was 70.6% (255 of 361 cases). A comprehensive summary of baseline patient characteristics is provided in Table 1.

### 3.2. Uni- and Multivariate Analyses

In the univariate analyses, multiple variables demonstrated significant correlations with SF outcomes (Table 2). Multivariate logistic regression analysis was conducted using variables that demonstrated statistical significance in univariate analysis, including history of urolithiasis, maximum stone size, lower-pole kidney stone, number of stones ≥ 3, CKD ≥ G3, urine WBC positivity, grade IV hydronephrosis, and mean CT attenuation value (Table 3). Because their component factors overlapped with other significant predictors, both the T.O.HO. and S.T.O.N.E. scores were not incorporated into the multivariable regression model.

### 3.3. ROC Curve Analysis

**Figure 1:**
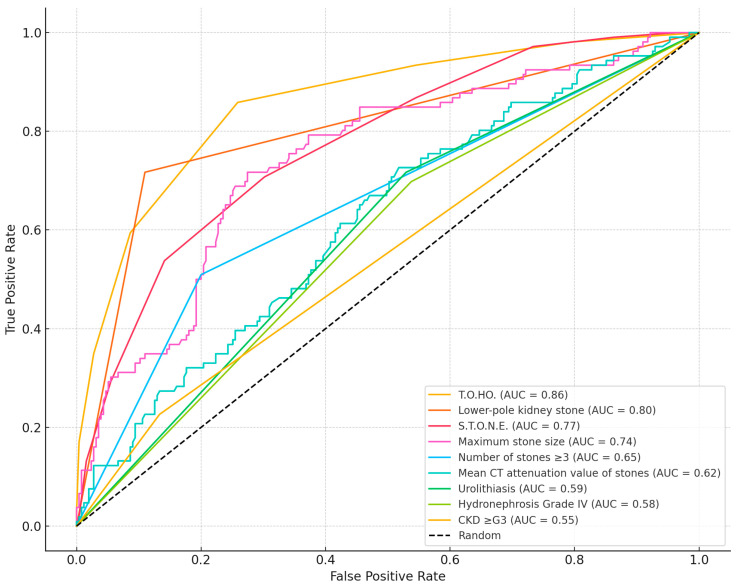
Comparison of the AUC values between T.O.HO. and S.T.O.N.E. score and each predictor.

To evaluate discriminatory ability, ROC curves were generated for both the T.O.HO. and S.T.O.N.E. scoring systems as well as for selected clinical predictors (Table 4, Figure 1). The T.O.HO. model showed the strongest performance in forecasting SF outcomes, with an AUC of 0.86 (95% CI: 0.82–0.90). This was significantly higher than the AUC obtained for the S.T.O.N.E. score (AUC: 0.77; 95% CI: 0.72–0.83; *p* < 0.01).

### 3.4. Calibration and DCA

**Figure 2:**
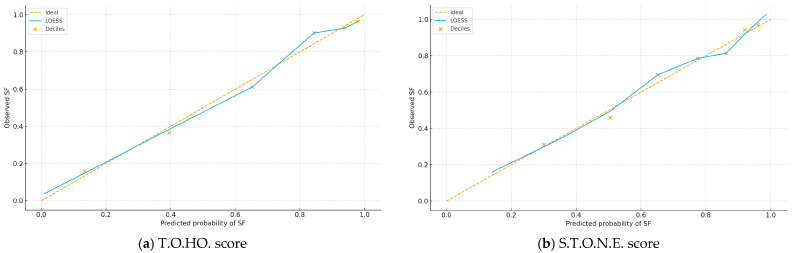
Calibration Plots of the T.O.HO. and S.T.O.N.E. Scores for Stone-Free Prediction After fURS.

**Figure 3:**
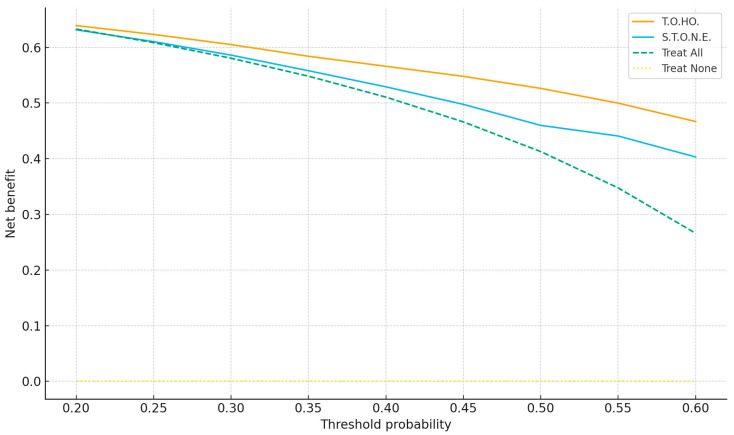
Decision-Curve Analysis Comparing T.O.HO. vs. S.T.O.N.E. scores.

Figure 2a,b plot observed versus predicted SF probabilities; curves close to the 45° line indicate good calibration, which was acceptable for both scores. Figure 3 shows decision curves; across thresholds 0.20–0.60, the T.O.HO. curve lies above S.T.O.N.E. and the “treat-all/none” strategies, indicating greater clinical utility and supporting pragmatic cut-off selection.

### 3.5. Threshold Metrics

We defined clinically meaningful cut-offs on the raw score scale using (i) Youden’s J and (ii) a high-sensitivity target (sensitivity ≥ 0.85). Because higher raw scores indicate greater risk of not achieving SF status, patients were classified as SF when the score was ≤ the cut-off. At the Youden threshold, T.O.HO. ≤ 5 yielded sensitivity 74.1%, specificity 85.9%, PPV 92.6%, and NPV 58.0%; S.T.O.N.E. ≤ 10 yielded 69.8%, 70.8%, 85.2%, and 49.3%, respectively. Using a high-sensitivity option, T.O.HO. ≤ 6 achieved 91.4%/59.4%/84.4%/74.1% (sensitivity/specificity/PPV/NPV), and S.T.O.N.E. ≤ 11 achieved 85.9%/53.8%/81.7%/61.3%.

## 4. Discussion

This external validation study confirms that the T.O.HO. score remains a robust and clinically meaningful tool to estimate SF status following fURS. Among 361 patients, the T.O.HO. score demonstrated excellent discriminative performance (AUC 0.86), surpassing both the S.T.O.N.E. score (AUC 0.77) and other individual clinical predictors. These findings are consistent with those of Noah and colleagues. (2025), who prospectively compared three nomograms—including the T.O.HO. and S.T.O.N.E. scores—and demonstrated that the T.O.HO. score provided significantly better discrimination for predicting stone-free outcomes (AUC 0.860 vs. 0.805, *p* < 0.05) [23]. In contrast, earlier studies assessing the S.T.O.N.E. score across diverse patient groups have generally shown only moderate predictive performance, with reported AUC values ranging between 0.617 and 0.725 [21,24,25].

The T.O.HO. score incorporates three objective, imaging-based parameters—stone length, stone location, and HU of stone—allowing for rapid and consistent application without reliance on complex reconstruction or subjective assessment [12]. Although the S.T.O.N.E. score is likewise based on CT-derived parameters and serves as a useful clinical instrument [11], the T.O.HO. score offers equivalent or superior predictive capacity with fewer variables. In contrast to more elaborate schemes such as the R.I.R.S. and H.L.P.E.S. scores, the T.O.HO. model is distinguished by its simplicity and suitability for routine clinical use, particularly in preoperative decision-making and patient counseling [9,10].

Our uni- and multivariate analyses identified several key predictors of postoperative SF status: a history of urolithiasis, maximum stone size, lower-pole renal stones, the presence of three or more stones, CKD ≥ Grade 3, positive urine WBC, hydronephrosis grade IV, and the mean CT attenuation value of stones. Notably, many of these variables—stone number, location, mean HU, and degree of obstruction—are already integrated into the T.O.HO. or S.T.O.N.E. score [11,12]. Previous studies have shown that increased stone number, size, lower-pole location, and higher CT attenuation values significantly reduce the likelihood of achieving a SF state [10,20,26,27,28,29,30].

Leveraging calibrated T.O.HO. probabilities and threshold-aware metrics, we propose risk-stratified counseling: ≤5 (high probability of single-session SF; proceed with standard fURS), 6 (intermediate; discuss trade-offs and consider adjunct repositioning and selective access-sheath use), and ≥7 (higher residual risk; counsel on staged procedures and plan resources proactively). Independent predictors (lower-pole location, stone size, urine WBC positivity) translate into actionable steps, including lower-pole repositioning and preoperative infection optimization. DCA demonstrates where adopting T.O.HO. confers greater net benefit than default strategies (thresholds 0.20–0.60), enabling shared decision-making aligned with patient preferences. We additionally present threshold-oriented metrics—Youden’s J–based and high-sensitivity cut-offs—to inform shared decision-making, thereby aligning individualized risk with pragmatic management (e.g., proceeding with single-session fURS vs. planning staged procedures).

Lower-pole renal calculi, in particular, emerged as a potent determinant of SF failure (Figure 1), likely owing to anatomical constraints such as limited accessibility and unfavorable angulation for fragmentation and retrieval. Clinically, patients with lower-pole stones should be counseled preoperatively about the increased likelihood of staged procedures. To optimize outcomes, adjunctive strategies—stone repositioning, the use of disposable flexible ureteroscopes, or instruments with enhanced deflection capability—may be considered. In addition, selective use of ureteral access sheaths can facilitate fragment retrieval and improve clearance.

Beyond these established factors, our study identified additional clinically relevant predictors. Preoperative pyuria was significantly associated with lower SF rates following fURS, likely reflecting subclinical infection or an inflammatory milieu that complicates endoscopic management. Infection-related stones such as struvite calculi, formed by urease-producing bacteria, are known to require complete eradication yet are often difficult to remove in a single session, thereby reducing one-session success rates [29,31]. We did not systematically analyze stone composition; thus, a direct causal link between pyuria and infection stones cannot be established. Even in the absence of confirmed infection, inflammation associated with pyuria may contribute to mucosal edema or ureteral wall thickening, impeding complete clearance. Multiple studies have also shown that preoperative pyuria correlates with increased intraoperative complexity and higher residual fragment rates [29,32,33]. Accordingly, pyuria should be regarded as a predictor of surgical difficulty and residual fragments rather than definitive evidence of infectious stones, and patients with preoperative pyuria may benefit from infection optimization, including culture-directed antibiotic therapy, prior to fURS.

Our univariate analysis also revealed that CKD ≥ Grade 3 was associated with a lower SF rate following fURS. Ma and colleagues identified decreased preoperative ipsilateral renal function as an independent risk factor for residual stone burden, suggesting that renal dysfunction may compromise clearance efficiency through anatomical or physiological mechanisms [34]. Similarly, Akdoğan and colleagues reported that individuals who underwent PCNL with an eGFR below 30 experienced markedly reduced stone-free rates compared with patients possessing preserved renal function [35]. In CKD patients, impaired urinary flow may reduce postoperative fragment washout [35,36], and chronic parenchymal changes—such as altered calyceal anatomy or reduced renal mobility—can hinder endoscopic access and visualization [35]. It is also important to differentiate true CKD from transient obstruction-induced renal impairment. Reeves et al. showed that eGFR significantly improved within six months after relief of obstruction by ureteroscopy, supporting the reversibility of post-renal acute kidney injury [37].

Furthermore, a history of urolithiasis was associated with poorer SF outcomes in univariate analysis. This may reflect underlying metabolic predisposition, anatomical anomalies prone to recurrence, or fibrotic changes from prior interventions. Indeed, a large multicenter study found that patients with previous stone procedures had nearly double the risk of requiring reintervention after fURS [38].

The clinical importance of achieving complete stone clearance cannot be overstated. Multiple studies have underscored that patients with residual fragments after fURS face a significantly increased risk of recurrence, necessitating repeat interventions, incurring greater healthcare costs, and potentially worsening renal function. Some researchers. reported that the five-year recurrence rate can reach 50%, particularly among patients with prior stone history, metabolic abnormalities, or incomplete clearance [39,40,41]. These findings highlight the need to pursue complete clearance during the initial procedure and to implement structured surveillance and secondary prevention strategies for high-risk patients. Our observed SF rate of 70.6% is consistent with recent fURS series using CT-based definitions. Although the detection of CIRFs is stringent under CT criteria, complete stone clearance remains the surgical gold standard to reduce recurrence and reintervention.It is worth noting that we did not evaluate the modified T.O.HO. score proposed by Polat and colleagues, which incorporates laterality and stone volume [21]. While such modifications may enhance predictive precision, they also introduce additional complexity. Our findings suggest that the original T.O.HO. score maintains excellent predictive accuracy and may offer practical advantages in busy clinical settings due to its simplicity.

Several limitations merit consideration. First, the retrospective, single-center design introduces the potential for selection and institutional biases (e.g., the requirement for postoperative NCCT within 3 months), which cannot be fully eliminated. Second, although this constitutes an external validation outside the derivation center, our cohort was accrued in Japan, where device availability, perioperative pathways, and surgical techniques likely resemble those in the derivation setting; such geographic/device/practice similarities may have inflated observed performance and limit generalizability. Third, outcome adjudication relied on CT-based parameters that are subject to interobserver variability, and routine stone composition analysis was not performed, precluding mechanistic inferences (e.g., infectious/struvite stones). Fourth, while we identified predictors not included in existing nomograms, we did not construct or recalibrate an integrated model incorporating these variables; the study was not designed or powered for model rebuilding. Finally, as part of this revision, we corrected typographical issues, harmonized abbreviations at first use, and standardized *p*-value and CI formatting across the text, tables, and figure legends in line with journal guidance. Prospective, multicenter—ideally international—validation across diverse institutions, platforms, and practice environments, coupled with standardized imaging protocols and central/consensus reading, will be essential to establish transportability and to refine threshold-guided, personalized fURS planning.

## 5. Conclusions

In conclusion, the T.O.HO. scoring system represents a reliable and practical approach for anticipating SF outcomes after fURS, providing valuable support for operative decision-making and improving the quality of patient counseling. Its simplicity and robust predictive accuracy render it an excellent candidate for routine incorporation into preoperative assessment protocols. By complementing discrimination with calibration and decision curve metrics, T.O.HO. functions as a personalized decision aid for fURS planning.

## Figures and Tables

**Table 1:** Baseline demographic and clinical characteristics of the study cohort.

Variables	Total (N = 361)
Sex (Male/Female), n (%)	228 (63.2)/133 (36.8)
Age (years), Median (IQR)	62.0 (52.0–73.0)
BMI (kg/m^2^), Median (IQR)	24.4 (21.8–26.9)
ECOG-PS ≥ 2, n (%)	12 (3.3)
Medical history:	
Urolithiasis, n (%)	211 (58.5)
Diabetes mellitus, n (%)	71 (19.7)
Hyperparathyroidism, n (%)	11 (3.0)
Medication use:	
α_1_-blocker	35 (9.7)
Anticoagulant/antiplatelet agents, n (%)	27 (7.5)
Corticosteroid, n (%)	8 (2.2)
Pre-stenting, n (%)	145 (40.2)
Ureteral stone characteristics:	
Maximum stone size (mm), Median (IQR)	10.1 (7.0–13.6)
Total stone length (mm), Median (IQR)	12.9 (8.3–19.4)
Laterality (Right/Left/Bilateral) , n (%)	181 (50.1)/160 (44.3)/20 (5.6)
Ureteral stone location (Upper/Middle/Lower), n (%)	206 (74.9)/37 (13.5)/32 (11.6)
Kidney stone location (Upper/Middle/Lower), n (%)	36 (21.1)/31 (18.1)/104 (60.8)
Number of stones (1/2/ ≥ 3)	169 (46.8)/87 (24.1)/105 (29.1)
Laboratory findings:	
WBC (/μL), Median (IQR)	7030 (5660–8980)
Hb (g/dL) , Median (IQR)	13.9 (12.8–15.0)
Platelet (×10^3^/μL), Median (IQR)	241 (195–279)
CRP (mg/dL), Median (IQR)	0.2 (0.1–1.7)
HbA1c (%), Median (IQR)	5.8 (5.5–6.3)
CKD ≥ G3	58 (16.1)
Urinalysis:	
Urine pH, Median (IQR)	6.0 (5.5–7.0)
Urine WBC (positive), n (%)	206 (57.4)
Urine nitrite (positive), n (%)	20 (5.6)
Radiographic findings:	
Hydronephrosis grade (None/Grade I/Grade II/Grade III/Grade IV), n (%)	128 (35.5)/ 96 (26.6)/ 93 (25.8)/ 37 (10.2)/ 7 (1.9)
Mean CT attenuation value of stones (HU), Median (IQR)	862.0 (584.0–1120.0)
Solitary kidney, n (%)	3 (0.8)
Operative time (min), Median (IQR)	77.0 (56.0–98.0)
T.O.HO. score, Median (IQR)	5.0 (4.0–6.0)
S.T.O.N.E. score, Median (IQR)	10.0 (9.0–12.0)
Stone-free rate, n (%)	255 (70.6)

BMI: body mass index, CKD: Chronic kidney disease, CRP: C-reactive protein, CT: computed tomography, ECOG-PS: Eastern Cooperative Oncology Group-Performance Status, Hb: hemoglobin, HU: Hounsfield units, IQR: interquartile range, WBC: white blood cells. Percentages for stone location were calculated among patients with ureteral or kidney stones.

**Table 2:** Univariate Analyses of Clinical Predictors for Stone-Free Status.

Variables	Stone-Free (n = 255)	Non-Stone-Free (n = 106)	*p* Value
Sex (Female), n (%)	97 (38.0)	36 (34.0)	0.48
Age (years), Median (IQR)	61.0 (51.0–72.0)	62.0 (54.0–74.0)	0.19
BMI (kg/m^2^), Median (IQR)	24.5 (22.0–27.1)	23.8 (21.3–26.6)	0.12
ECOG-PS ≥ 2, n (%)	9 (3.5)	3 (2.8)	1.00
Medical history:			
Urolithiasis, n (%)	135 (52.9)	76 (71.6)	<0.01
Diabetes mellitus, n (%)	55 (21.6)	16 (15.1)	0.19
Hyperparathyroidism, n (%)	5 (2.0)	6 (5.7)	0.09
Medications use:			
α_1_-blocker	22 (8.6)	13 (12.3)	0.33
Anticoagulant/antiplatelet agents, n (%)	17 (6.7)	10 (9.4)	0.36
Corticosteroid, n (%)	4 (1.6)	4 (3.8)	0.18
Pre-stenting, n (%)	110 (43.1)	35 (33.0)	0.07
Ureteral stone characteristics:			
Maximum stone size (mm), Median (IQR)	8.5 (6.5–11.6)	12.9 (10.6–15.0)	<0.01
Total stone length (mm), Median (IQR)	11.0 (7.5–15.7)	19.1 (13.0–25.0)	<0.01
Laterality (Right/Left/Bilateral), n (%)	126 (49.4)/117 (45.9)/12 (4.7)	55 (51.9)/43 (40.6)/8 (7.5)	0.44
Ureteral stone location (Upper/Middle/Lower), n (%)	161 (74.2)/29 (13.4)/27 (12.4)	45 (77.6)/8 (13.8)/5 (8.6)	0.16
Kidney stone location (Upper/Middle/Lower), n (%)	26 (34.7)/21 (28.0)/28 (37.3)	10 (10.4)/10 (10.4)/ 76 (79.2)	<0.01
The number of stones (1/2/ ≥ 3), n (%)	140 (54.7) /64 (25.0) /51 (20.3)	29 (27.4)/23 (21.7)/54 (50.9)	<0.01
Laboratory findings:			
WBC (/μL), Median (IQR)	7050 (5615–9320)	6785 (5700–8282)	0.15
Hb (g/dL) , Median (IQR)	13.9 (12.7–15.0)	14.1 (13.1–15.2)	0.25
Platelet (×10^3^/μL), Median (IQR)	243 (194–277)	238 (201–286)	0.72
CRP (mg/dL), Median (IQR)	0.29 (0.06–2.02)	0.14 (0.04–0.84)	0.09
HbA1c (%), Median (IQR)	5.8 (5.5–6.4)	5.8 (5.5–6.1)	0.16
CKD ≥ G3	34 (13.3)	24 (22.6)	0.04
Urinalysis			
Urine pH	6.0 (5.5–7.0)	6.5 (6.0–7.0)	0.22
Urine WBC (positive), n (%)	133 (52.6)	73 (68.9)	<0.01
Urine nitrite (positive), n (%)	15 (5.9)	5 (4.8)	0.80
Radiographic findings:			
Hydronephrosis grade (None/Grade I/Grade II/Grade III/Grade IV) , n (%)	76 (29.8)/79 (30.9)/70 (27.5)/27 (10.6)/3 (1.2)	52 (49.1)/17 (16.0)/23 (21.7)/10 (9.4)/4 (3.8)	<0.01
Mean CT attenuation value of stones (HU), Median (IQR)	816.0 (551.0–1076.0)	963.5 (763.0–1231.8)	<0.01
Solitary kidney, n (%)	2 (0.8)	1 (0.9)	1.00
Operative time (min), Median (IQR)	71.0 (48.0–91.5)	93.0 (72.0–114.8)	<0.01
T.O.HO. score, Median (IQR)	5.0 (4.0–6.0)	7.0 (6.0–8.0)	<0.01
S.T.O.N.E. score, Median (IQR)	10.0 (8.0–11.0)	12.0 (10.0–13.0)	<0.01

BMI: body mass index, CKD: Chronic kidney disease, CRP: C-reactive protein, CT: computed tomography, ECOG-PS: Eastern Cooperative Oncology Group-Performance Status, Hb: hemoglobin, HU: Hounsfield units, IQR: interquartile range, WBC: white blood cells.

**Table 3:** Multivariate Analysis of Clinical Predictors for Stone-Free Status.

Variables	Adjusted Odds Ratio (95% CI)	*p* Value
Medical history of Urolithiasis	1.08 (0.55–2.11)	0.82
Maximum stone size (Per 1.0 mm)	1.15 (1.08–1.22)	<0.01
Per 5.0 mm	1.99 (1.45–2.74)	
Per 10.0 mm	3.94 (2.09–7.46)	
Lower-pole kidney stone	19.09 (9.34–39.01)	<0.01
Number of stones ≥ 3	1.89 (0.96–3.74)	0.07
CKD ≥ G3	1.29 (0.55–3.01)	0.56
Urine WBC (positive)	2.25 (1.14–4.44)	0.02
Hydronephrosis Grade IV	0.80 (0.41–1.55)	0.50
Mean CT attenuation value of stones	1.00 (1.00–1.00)	0.05

CI: confidence interval, CKD: chronic kidney disease, CT: computed tomography, WBC: white blood cell.

**Table 4:** Area Under the Curve Values for Predictive Models.

Clinical Predictors	AUC (95% CI)	*p* Value (Compared with T.O.HO. Score)
T.O.HO. score	0.86 (0.82–0.90)	-
S.T.O.N.E score	0.77 (0.72–0.83)	<0.01
Lower-pole kidney stone	0.80 (0.75–0.86)	0.14
Maximum stone size	0.74 (0.68–0.80)	<0.01
Number of stones ≥ 3	0.65 (0.59–0.72)	<0.01
Mean CT attenuation value of stones	0.62 (0.55–0.68)	<0.01
Medical history of Urolithiasis	0.59 (0.53–0.66)	<0.01
Urine WBC	0.58 (0.52–0.65)	<0.01
Hydronephrosis Grade IV	0.58 (0.52–0.64)	<0.01
CKD ≥ G3	0.55 (0.48–0.61)	<0.01

CKD: chronic kidney disease, CT: computed tomography, WBC: white blood cell.

## Data Availability

The data presented in this study are available on request from the corresponding author due to ethical concerns.

## Abbreviations

The following abbreviations are used in this manuscript:

AUC	area under the curve
BMI	body mass index
CI	confidence interval
CKD	chronic kidney disease
CRP	C-reactive protein
CT	computed tomography
DCA	decision curve analysis
ECOG-PS	Eastern Cooperative Oncology Group–Performance Status
eGFR	estimated glomerular filtration rate
fURS	flexible ureteroscopy
Hb	hemoglobin
HbA1c	hemoglobin A1c
HU	Hounsfield units
IQR	interquartile range
LOESS	locally estimated scatterplot smoothing
NCCT	non-contrast computed tomography
NPV	negative predictive value
OR	odds ratio
PPV	positive predictive value
ROC	receiver operating characteristic
SF	stone-free
WBC	white blood cell
